# Comparison of 3 Randomized Clinical Trials of Frontline Therapies for Malignant Pleural Mesothelioma

**DOI:** 10.1001/jamanetworkopen.2022.1490

**Published:** 2022-03-09

**Authors:** Tomer Meirson, Francesca Pentimalli, Francesco Cerza, Giovanni Baglio, Steven G. Gray, Pierpaolo Correale, Marija Krstic-Demonacos, Gal Markel, Antonio Giordano, David Bomze, Luciano Mutti

**Affiliations:** 1 Davidoff Cancer Center, Rabin Medical Center–Beilinson Hospital, Petah Tikva, Israel; 2 Dipartimento di Medicina e Chirurgia, Libera Università Mediterranea “Giuseppe Degennaro”, Bari, Italy; 3 Italian National Agency for Regional Healthcare Services, Rome; 4 Thoracic Oncology Research Group, Trinity St James’s Cancer Institute, Trinity Translational Medicine Institute, Trinity Centre for Health Sciences, St James’s Hospital, Dublin, Ireland; 5 Unit of Medical Oncology, Oncology Department, Grand Metropolitan Hospital “Bianchi Melacrino Morelli,” Reggio Calabria, Italy; 6 Sbarro Institute for Cancer Research and Molecular Medicine, Center for Biotechnology, College of Science and Technology, Temple University, Philadelphia, Pennsylvania; 7 Biomedical Research Center, School of Science, Engineering and Environment, University of Salford, Salford, United Kingdom; 8 Sackler Faculty of Medicine, Tel Aviv University, Tel Aviv, Israel; 9 Department of Medical Biotechnologies, University of Siena, Siena, Italy

## Abstract

**Importance:**

Some recently proposed frontline therapies for malignant pleural mesothelioma (MPM) are very costly, yet their impact on quality of life and overall survival of these patients remains arguable. Given the high social toll of this aggressive occupational cancer, it is paramount to establish the real clinical benefit of these treatments.

**Objective:**

To directly compare and analyze the statistical robustness of the 3 randomized clinical trials (RCTs) of frontline therapies recommended for MPM since 2003.

**Design, Setting, and Participants:**

This comparative effectiveness study assessed the following phase 3 RCTs: the Mesothelioma Cisplatin Pemetrexed Study (MPS) of cisplatin plus pemetrexed vs cisplatin; the Mesothelioma Avastin Cisplatin Pemetrexed Study (MAPS) of cisplatin plus pemetrexed plus bevacizumab vs cisplatin plus pemetrexed; and the CheckMate743 (CM743) study of nivolumab plus ipilimumab vs cisplatin plus pemetrexed. Data collection dates for the RCTs ranged from April 1999 to April 2018. Data for this study were analyzed from February to October 2021.

**Main Outcomes and Measures:**

Patient selection criteria, superiority of the intervention groups, survival-inferred fragility index, and censoring patterns in each RCT.

**Results:**

A total of 1501 patients were included in the analysis (1170 men [77.9%]; range of median age for treatment groups, 60 [IQR, 19-84] to 69 [IQR, 65-75] years). A virtual comparison of overall survival in MAPS vs the CM743 study showed no statistically significant difference (hazard ratio [HR], 0.97 [95% CI, 0.79-1.20]; *P* = .79), and the survival-inferred fragility index in the intention-to-treat (ITT) populations was as low as 0.22% of the total sample size in MPS, −0.45% of the total sample size in MAPS, and 0.99% of the total sample size in the CM743 trial. Moreover, reverse restricted mean survival time (RMST) analysis of overall survival using RMST-difference (RMST-D) demonstrated differential censoring in the ITT population of the CM743 trial favoring the control group (0.56 [95% CI, 0.18-0.94]; *P* = .004) and in the nonepithelioid group (reverse RMST-D, 0.90 [95% CI, 0.001-1.79]; *P* = .048).

**Conclusions and Relevance:**

This comparative effectiveness study found no survival benefit in the CM743 trial over MAPS, despite the inclusion of patients with worse prognosis in the latter trial. Moreover, the statistical conclusions of all the examined trials were shown to be extremely fragile, and the findings of differential censoring in the CM743 trial and in the ITT nonepithelial subset raised additional areas of concern. These findings suggest that selection criteria, fragility, and censoring patterns may affect the original conclusions drawn for the respective trials, casting a shadow on the real benefit. This model of analysis lays a rigorous groundwork extendable to trials of all cancer treatments before their registration.

## Key Points

**Question:**

Are frontline therapies recommended for malignant pleural mesothelioma (MPM) associated with patient benefits?

**Findings:**

In this comparative effectiveness study of 1501 participants, the statistical robustness of the Mesothelioma Cisplatin Pemetrexed Study (MPS), Mesothelioma Avastin Cisplatin Pemetrexed Study (MAPS), and CheckMate743 (CM743) study were assessed by analysis of the survival curves, survival-inferred fragility index, and censoring patterns. The overall survival curves of the CM743 trial and MAPS overlapped, despite worse performance status in MAPS; unequal censoring was observed in the CM743 trial, particularly in the nonepithelioid subtype, and the survival-inferred fragility index was low in all trials.

**Meaning:**

These findings suggest that no conclusion regarding survival benefit can be drawn from the 3 included trials; future clinical trials involving novel cancer treatments should consider more factors before registration.

## Teaser

This comparative effectiveness study analyzes the designs, conduct, and outcomes of the Mesothelioma Cisplatin Pemetrexed Study, Mesothelioma Avastin Cisplatin Pemetrexed Study, and CheckMate743 clinical trial to achieve a better understanding of the association of the studied therapies with clinical outcomes among patients with malignant pleural mesothelioma.

## Introduction

Malignant pleural mesothelioma (MPM) is the primary cancer of the pleural lining; it is heavily associated with exposure to asbestos fibers and affected more than 30 000 patients globally in 2020.^1^ Prognosis is poor (mean survival, approximately 12 months from diagnosis), and current therapies have only modestly improved patient survival.^2^ Because of this bleak scenario, MPM has been designated an “orphan disease” by the European Medicines Agency,^3^ and there is an urgent need to identify effective therapeutic strategies.

Since 2003, 3 different approaches tested via phase 3 randomized clinical trials (RCTs) have been subsequently considered frontline therapies for unresectable MPM, each of which was proposed to replace the previous therapy because of presumed better performance. First, doublet chemotherapy with cisplatin and pemetrexed (the Mesothelioma Cisplatin Pemetrexed Study [MPS])^4^ was replaced by triplet cisplatin and pemetrexed plus the antiangiogenic bevacizumab (the Mesothelioma Avastin Cisplatin Pemetrexed Study [MAPS]),^2,5^ followed by the combined immune check point inhibitors nivolumab plus ipilimumab (CheckMate743 [CM743]),^6^ which has been proposed in lieu of the first two and recently approved by the US Food and Drug Administration.^7^ The limited increases in survival benefit for each of these treatments prompted us to perform a deeper analysis of the designs, conduct, and outcomes of these 3 RCTs to achieve a better understanding of how they truly benefit patients with MPM.

## Methods

### Study Design

In this comparative effectiveness study, we analyzed data from all 3 major phase 3 RCTs of frontline therapy for MPM: MPS (performed from April 1999 to March 2001; median follow-up, 10.0 months [IQR not available]),^4^ MAPS (performed from February 2008 to January 2014; median follow-up, 39.4 [IQR, 25.5-54.8] months),^5^ and CM743 (performed from November 2016 to April 2018; median follow-up, 29.7 [IQR, 26.7-32.9] months).^6^ The basic characteristics of the patients enrolled in the selected studies are reported in the eTable in the Supplement. The clinical studies were approved by their respective institutional review boards, and all patients provided written informed consent. Because the study used anonymized records and deidentified data sets that exist in the public domain, no further ethics committee approval was required. For the purposes of our study, we followed the International Society for Pharmacoeconomics and Outcomes Research (ISPOR) reporting guideline where applicable.

### Statistical Analysis

#### Survival Analysis

Data were analyzed for this study from February to October 2021. The Kaplan-Meier curves were extracted using WebPlotDigitizer, version 4.3, and reconstructed using the reconstruct KM package in R, version 0.1.0 (R Project for Statistical Computing). This reconstruction strategy enables the reproduction of time-to-event data at the individual patient level with minor differences between the reconstructed and original data. The follow-up time distribution was estimated using the prodlim package in R, version 2019.11.13.

#### Survival-Inferred Fragility Index Calculation

The survival-inferred fragility index (SIFI) was calculated from the Kaplan-Meier curves by the iterative redesignation of the participants with the longest survival from the intervention to the control group until positive significance was lost.^8^ We used the boundary for significance as specified in the statistical design for each study: *P* = .0476 for MPS; *P* = .029 for MAPS; and *P* = .0345 for CM743. For the nonepithelioid group, no statistical boundary was defined. Although the boundary is expected to be lower, we used the same nominal value as the intention-to-treat (ITT) population of the CM743 study. We performed the statistical analysis using a 2-sided unstratified log-rank test.

#### Censoring Analysis

The reverse Kaplan-Meier curves were generated by flipping the status of the time-dependent outcome (ie, 1 indicates censored; 0, death). The Cox proportional hazards modeling was performed using the survival package in R, version 3.2-7, on the reverse Kaplan-Meier curves to compare the censoring rates and calculate the reverse hazard ratio (HR). The restricted mean survival time (RMST) is the nonparametric alternative strategy of the HR that does not rely on the proportional hazards assumption.^9,10^ The RMST difference (RMST-D), the area bounded by 2 Kaplan-Meier curves, reflects the absolute gain or loss in survival. Therefore, we also calculated the analogous reverse RMST-D using the survRM2 package in R, version 1.0-3. The reverse RMST-D was truncated at half of the follow-up time to assess early censoring imbalances. *P* values of the treatment effects (standard Kaplan-Meier) and censoring imbalances (reverse Kaplan-Meier) were calculated using the 2-sided unstratified log-rank and RMST tests, with *P* < .05 indicating statistical significance.^11^

## Results

### Patients’ Features and Survival Analysis

A total of 1501 patients were included in the analysis (1170 men [77.9%] and 331 women [22.1%]; range of median age for treatment groups, 60 [IQR, 19-84] to 69 [IQR, 65-75] years). Patient characteristics are summarized in the eTable in the Supplement; differences exist across the studies in the recruitment or grouping of patients with regard to performance status or histologic subtype, which are discussed below.

**Figure 1.**
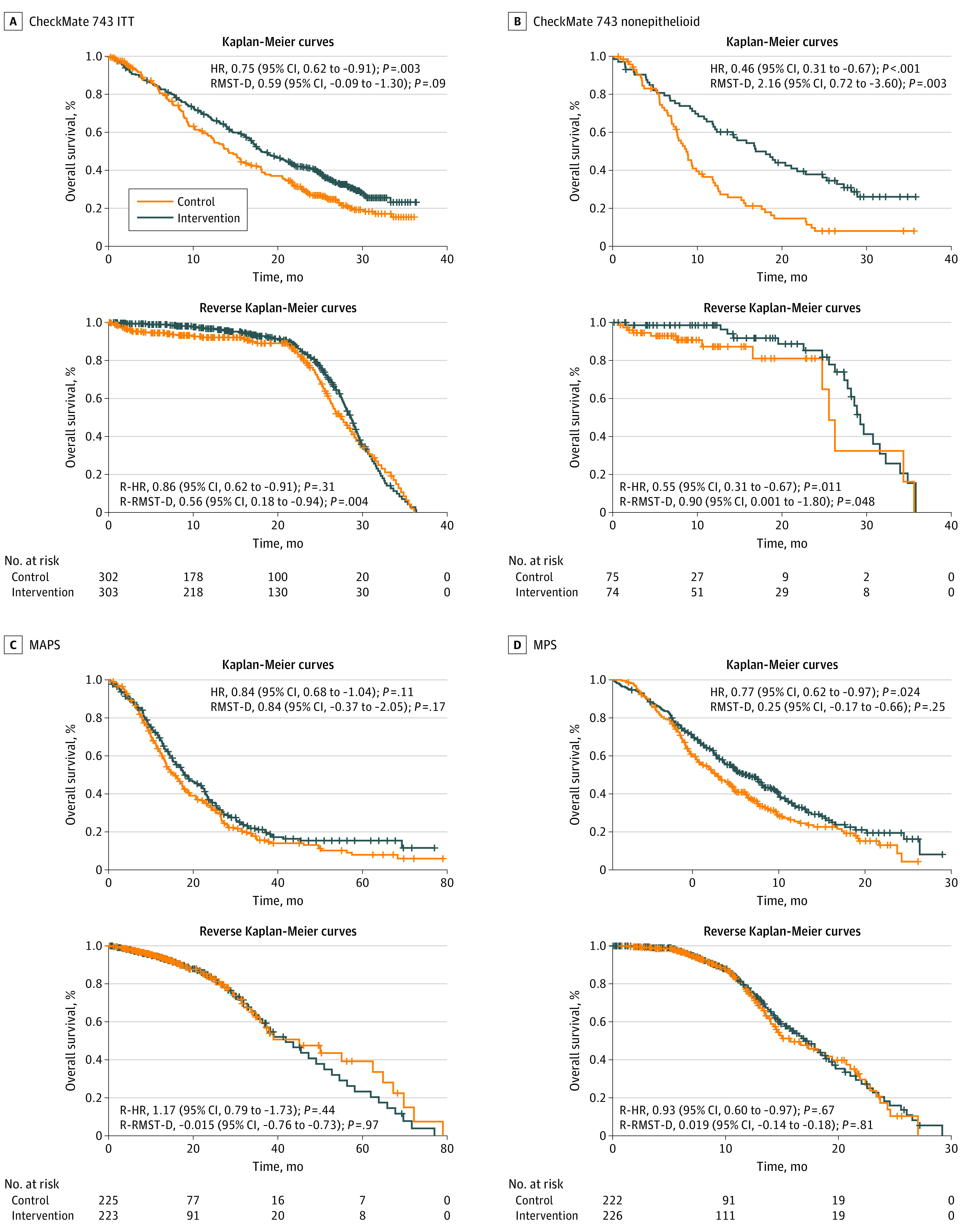
Differential Censoring Analysis of the 3 Frontline Phase 3 Randomized Clinical Trials Censoring analyses for overall survival in each study are shown. In the reverse Kaplan-Meier survival analysis, the status of the time-dependent outcome for individual patients is flipped. Censoring is considered the event of interest (ie, pseudoevent) and the original event as censored (ie, pseudo-censoring). HR indicates hazard ratio; MAPS, Mesothelioma Avastin Cisplatin Pemetrexed Study; MPS, Mesothelioma Cisplatin Pemetrexed Study; R-HR, reverse HR; RMST-D, restricted mean survival time–difference; and R-RMST-D, reverse RMST-D.

**Figure 2.**
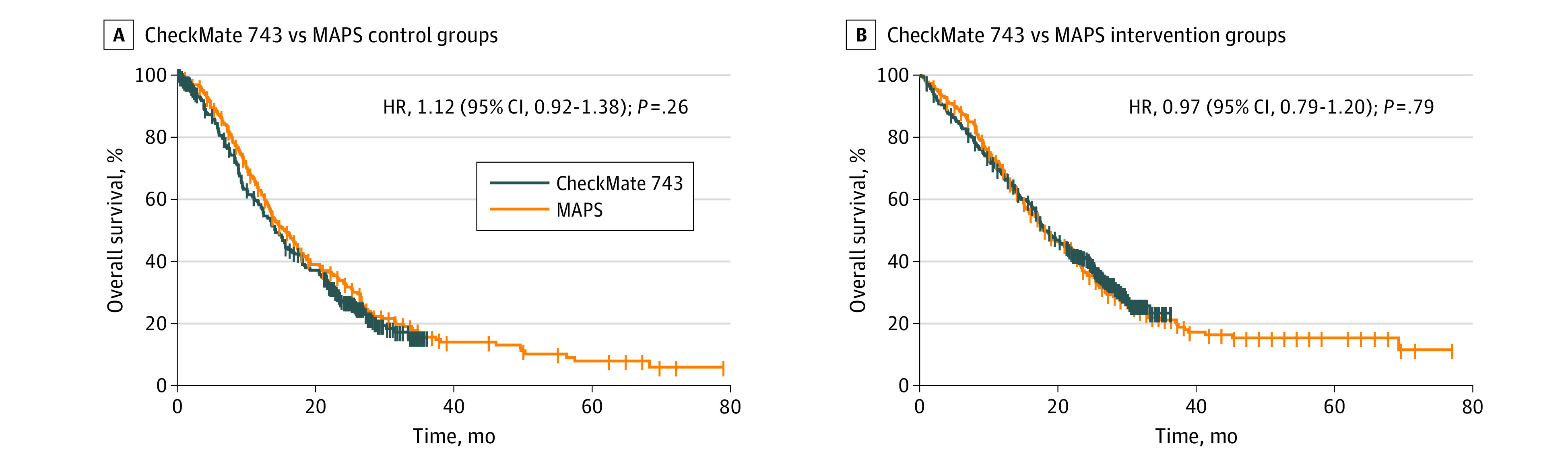
Virtual Head-to-Head Comparison of CheckMate743 and Mesothelioma Avastin Cisplatin Pemetrexed Study (MAPS) Studies Curves show virtual comparisons between the reconstructed overall survival curves of control and intervention groups. HR indicates hazard ratio.

We reconstructed the overall survival curves from the 3 RCTs (Figure 1). The control groups of the MAPS and CM743 trials overlapped and showed no significant differences; thus, in the virtual trial we were able to compare the intervention groups (Figure 2). The head-to-head comparison between nivolumab plus ipilimumab and cisplatin plus pemetrexed plus bevacizumab showed no statistically significant difference (HR, 0.97 [95% CI, 0.79-1.20]; *P* = .79). The recent report of 3-year survival of patients in the CM743 trial^12^ has provided similar results.

The difference between the calculated and reported HRs for overall survival is low (mean difference, 0.02 [range, 0-0.07]), indicating that the reconstruction methodology is accurate. However, because this study used unadjusted Cox proportional hazards models and log-rank tests, differences still exist. Notably, the statistical significance of the reconstructed patient-level overall survival data of MAPS was lost (HR, 0.84 [95% CI, 0.68-1.04]; *P* = .11) (Figure 1C). Moreover, only the nonepithelioid histologic subtype demonstrated a statistically significant increase in overall survival in the CM743 study (HR, 0.46 [95% CI, 0.31-0.67]; *P* < .001) (Figure 1B), so it greatly restricts the number of patients who could benefit from this combination.

### Survival-Inferred Fragility Index

To assess the fragility of the survival data, the SIFI^8^ was calculated for the studies with statistically significant overall survival benefit (eFigure in the Supplement). The SIFI values were 6 for the CM743 trial (and 6 for the nonepithelioid subtype), −2 for MAPS, and 1 for MPS. The SIFI in the ITT populations represents 0.99% of the total sample size of the CM743 trial, −0.45% of the total sample size of MAPS, and 0.22% of the total sample size of MPS. Therefore, the low SIFI of these trials does not allow us to state that any of the intervention groups improve patients’ survival with high statistical certainty.

### Censoring Analysis

Censoring analysis is included in Figure 1. Significant differential censoring was observed in the ITT population of the CM743 trial, favoring the control group using the reverse RMST-D analysis (0.56 [95% CI, 0.18-0.94]; *P* = .004). In addition, the nonepithelioid group was associated with a significant censoring imbalance in the control group (reverse RMST-D, 0.90 [95% CI, 0.001-1.80]; *P* = .048).

## Discussion

Our Kaplan-Meier reconstructions show that the overall survival in the CM743 and MAPS intervention groups did not differ significantly (Figure 1), suggesting no superiority of one treatment over the other. Also, the results of these 2 treatments were associated with a low SIFI, as were those of the MPS (SIFI values of <1%).

The statistical significance of the reconstructed overall survival among MAPS participants was lost before calculating the SIFI, hence the negative value (SIFI, −2). This could be explained by our use of the unstratified log-rank test, which is less powerful than the adjustment based on the minimization variables used in MAPS. However, in most cases, the statistical conclusions of phase 3 oncology trials were not affected by changing the original design to an unstratified analysis.^8,11^ This finding suggests sensitivity of the MAPS results to the statistical modeling.

Importantly, the low SIFI in the ITT population means that only a small change in the randomization of the population could overturn the statistical conclusions. In other words, for all 3 treatments considered, if we were to switch 1% or fewer of the patients with better prognosis from the intervention group to the control group, the conclusions of the trial could change. Results with high fragility suggest poor robustness and uncertainty regarding the potential clinical benefit. Nonetheless, it is important to note that the SIFI does not reflect random variations in the assignment of individuals but the reassignment of the patients with the longest survival. Although reassignment of the 6 patients with the longest survival is much less likely than 6 random patients, in the CM743 trial this number represents a small fraction of the population (0.99%), and small variations in the randomization of patients with the best or worst prognosis is not unlikely. For example, small differences exist in the histologic subtypes of the CM743 trial (75% vs 76% for the epithelioid subtype and 25% vs 24% for the nonepithelioid subtype in the control and intervention groups, respectively).

The Kaplan-Meier method relies on the assumption of noninformative censoring, meaning that censoring is unrelated to the risk of the event. We used the reverse Kaplan-Meier method (ie, events and censoring are flipped) to focus on censoring patterns over time and examine differential censoring using the same standard statistical tools.^11^

Because we found significant censoring imbalances in the overall survival in the CM743 trial, the assumptions of the survival analysis were violated, which raises additional concerns regarding the conclusions of the study, particularly in the nonepithelioid subgroup (the only subgroup shown with improvement over the previous treatments) (Figure 1B). Nevertheless, evidence of unequal censoring between groups does not necessarily indicate that censoring must be related to survival time in the same way that equal censoring is not a validation of noninformative censoring. Moreover, interpretation of censoring favoring the control group is more complicated than that of the intervention group.^11^ However, informative censoring can only be analyzed through assessment of censoring imbalance when access to additional patient data is lacking. In any case, study investigators need to address potential biases relating to censored patients, especially when signs of informative censoring exist.^13^ Overall, these potential systemic biases in all 3 studies raise uncertainty regarding the robustness of the results and require a detailed investigation.

This study also raised additional concerns. The impact of performance status and histologic subtype on survival of patients with MPM is paramount for any treatment^14,15,16,17^; therefore, the absence of patients with performance status of 2 in the CM743 trial and the inclusion of nonepithelioid cases in MAPS should be also more carefully analyzed before declaring CM743 superiority.

Safety and financial expense of the therapies raise further distinct concerns. It is noteworthy that drug toxicity leading to discontinuation was reported in 59 of 300 of patients (19.7%) in the nivolumab plus ipilimumab group compared with 24 of 284 patients (8.5%) in the chemotherapy group.^6^

The cost-effectiveness ratio of approved immune checkpoint inhibitors for several human tumors is still debated. The estimated drug cost for cisplatin plus pemetrexed is $46 225 for 6 cycles, whereas the US patent on bevacizumab expired in 2019 and its European patent expires in 2022. Conversely, the combined cost for nivolumab plus ipilimumab is approximately $153 800 for 4 cycles,^18^ further stressing the need to consider the best treatment options with greater care. Such high-cost cancer drugs are currently being appraised by national health technology assessment agencies such as the Institute for Clinical and Economic Review and National Institute for Health and Care Excellence to determine whether they represent a cost-effective use of public resources measuring the cost per quality-adjusted life-year. A recent analysis^19^ showed that National Institute for Health and Care Excellence recommended 10 drugs for coverage by the national health system, but all 10 drugs included a financial agreement to improve cost-effectiveness and 7 were subject to additional evidence for efficacy. The present study emphasizes the need for more RCTs and real-world evidence for clinical decision-making policies.^19^ This is especially true for the nonepithelioid group in CM743, which was not prespecified in the protocol and should be considered hypothesis generating.

### Limitations

Our analysis was limited by the lack of access to complete patient characteristics of each individual enrolled in the trials, which should be provided to allow more accurate censoring evaluation and subgroup analysis to compare patients with the same performance status or histologic subtype, considering their well-established effect on patients’ survival. Previous studies have indeed emphasized differences between the highly select trial populations and real-world patients.^20^ Similarly, the time from diagnosis should be considered, given its association with patients’ survival.^14^ When considering the potential superiority of the CM743 over MAPS interventions, direct head-to-head comparisons in double-blind RCTs are needed and are more important than other statistical investigations such as censoring or fragility indices. Our statistical analysis is subject to fragility as well. Ideally, academic trials run by not-for-profit organizations could help to reduce potential biases that often characterize sponsored trials.^21^

## Conclusions

To our knowledge, this comparative effectiveness study is the first to compare the 3 clinical trials that have informed the frontline treatment paradigm of patients with MPM. Our findings suggest that no conclusion regarding survival benefit of the treatments proposed (or approved) can be drawn from the 3 included trials. We propose that a more careful assessment of any new cancer treatment (not only for MPM) should be performed in the future to assess its real impact before its approval for use in clinical settings.

## Notes

Supplement. **eTable.** Main Baseline Features of the Patients With MPM Enrolled in the 3 Frontline Phase 3 RCTs **eFigure.** Example of SIFI Calculation From the Reconstructed CheckMate743
