# Supplementary material for: Comparison of 3 Randomized Clinical Trials of Frontline Therapies for Malignant Pleural Mesothelioma
Source: JAMA Netw Open. 2022 Mar 9;5(3):e221490. doi: 10.1001/jamanetworkopen.2022.1490 (PMC8908075; doi:10.1001/jamanetworkopen.2022.1490)
Supplement: Supplement. — eTable. Main Baseline Features of the Patients With MPM Enrolled in the 3 Frontline Phase 3 RCTs eFigure. Example of SIFI Calculation From the Reconstructed CheckMate743 [file jamanetwopen-e221490-s001.pdf]

Supplementary Online Content

Meirson T, Pentimalli F, Cerza F, et al. Comparison of 3 randomized clinical trials of frontline therapies for malignant pleural mesothelioma. *JAMA Netw Open*. 2022;5(3):e221490. doi:10.1001/jamanetworkopen.2022.1490

eTable. Main Baseline Features of the Patients With MPM Enrolled in the 3 Frontline Phase 3 RCTs

eFigure. Example of SIFI Calculation From the Reconstructed CheckMate743

This supplementary material has been provided by the authors to give readers additional information about their work.

© 2022 Meirson T et al. *JAMA Network Open*.

eTable. Main Baseline Features of the Patients With MPM Enrolled in the 3 Frontline Phase 3 RCTs

Phase III RCT	MPS[¥]		MAPS		CheckMate 743	
	Pemetrexed plus Cisplatin	***Cisplatin***	***Pemetrexed plus cisplatin plus bevacizumab***	***Pemetrexed plus cisplatin***	***Nivolumab plus ipilimumab***	***Chemotherapy (Pemetrexed plus cisplatin or carboplatin)***
**Sex**						
Males	184 (81.4%)	181 (81.5%)	168 (75%)	170 (76%)	234 (77%)	233 (77%)
Females	42 (18.6%)	41 (18.5%)	55 (25%)	55 (24%)	69 (23%)	69 (23%)
**Median Age (Range)**						
	61 (29–85)	60 (19–84)	65.7 (61.5–70.0)	65.6 (60.8–70.3)	69 (65–75)	69 (62–75)
**Performance status**<br>***Scale; number of patients, (%) of patients***						
	Karnofsky ≥70<br>37 (16.4%)	Karnofsky ≥70<br>31 (14.0%)	ECOG 0–1<br>216 (97%)	ECOG 0–1<br>217 (96%)	ECOG 0<br>114 (38%)	ECOG 0<br>128 (42%)
	Karnofsky 80<br>72 (31.9%)	Karnofsky 80<br>66 (29.7%)	ECOG 2<br>7 (3%)	ECOG 2<br>8 (4%)	ECOG 1<br>189 (62%)	ECOG 1<br>173 (57%)
	Karnofsky 90/100<br>117 (51.8%)	Karnofsky 90/100<br>125 (56.3%)				
**Histology**						
Epithelioid	154 (68.1%)	152 (68.5%)	179 (80%)	182 (81%)	229 (76%)	227 (75%)
Sarcomatoid†	18 (8.0%)	25 (11.3%)	44 (20%)	43 (19%)	35 (12%)	36 (12%)
Mixed‡	37 (16.4%)	36 (16.2%)			39 (13%)	39 (13%)
Non-epithelioid					74 (24%)	75 (25%)
Unspecified	17 (7.5%)	9 (4.1%)				
**Stage**						
I	16 (7.1%)	14 (6.3%)	NA	NA	12 (4%)	20 (7%)
II	35 (15.6%)	33 (15.0%)	NA	NA	23 (8%)	22 (7%)
III	73 (32.4%)	68 (30.9%)	NA	NA	103 (34%)	106 (35%)
IV	102 (45.1%)*	107 (48.2%)*	NA	NA	160 (53%)	149 (49%)
Not reported					5 (2%)	5 (2%)

© 2022 Meirson T et al. *JAMA Network Open*.

ECOG: Eastern Cooperative Oncology Group; NA: not available; RCT: randomized clinical trial.

†The ‘sarcomatoid and mixed’ classes are reported jointly in the MAPS Study.

‡The Mixed class includes also ‘other’ variants in the Checkmate 743 Study.

*Includes patients with unspecified stage (one patient in pemetrexed/cisplatin arm and two patients in cisplatin arm).

¥For the MPS trial values from the intention to treat group are reported.

© 2022 Meirson T et al. *JAMA Network Open*.

eFigure. Example of SIFI Calculation From the Reconstructed CheckMate743

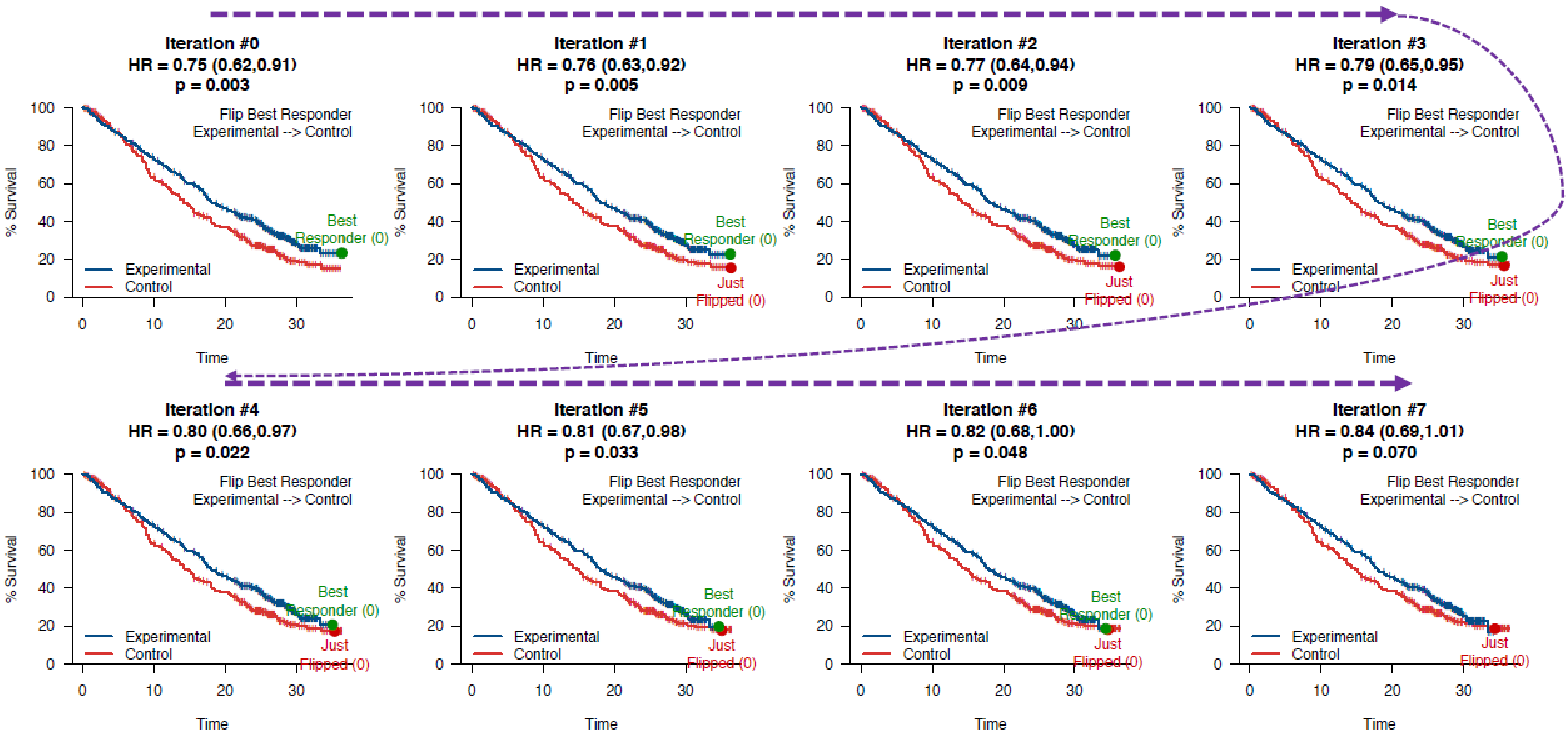

© 2022 Meirson T et al. *JAMA Network Open*.

Shown are the iterations of the survival-inferred fragility index (SIFI) for overall survival in the intention-to-treat population of CheckMate 743. In this example the SIFI is 7 which is the iterative reassignment of the longest survivors (designated by circles at the end of the survival curves) from the experimental group to the control group, until positive significance is lost (defined as α = .05 using log-rank test). HR indicates hazard ratio.

Reconstructed survival curve. B, Second iteration of the survival curve. C, Third iteration of the survival curve.

© 2022 Meirson T et al. *JAMA Network Open*.
